# The use of illustrated medication diaries to improve outcomes for children initiated on highly active antiretroviral therapy

**DOI:** 10.4102/sajhivmed.v19i1.804

**Published:** 2018-05-23

**Authors:** Yashodhara Kannigan, Kevin B. Spicer, Fathima Naby

**Affiliations:** 1 Department of Paediatrics and Child Health, Nelson R. Mandela School of Medicine, University of KwaZulu-Natal, South Africa; 2 Department of Paediatrics and Child Health, Pietermaritzburg Metropolitan Hospital Complex, South Africa

## Abstract

**Background:**

Human immunodeficiency virus (HIV) represents a huge burden of disease in South Africa. Highly active antiretroviral therapy (HAART) is effective in reducing HIV-related morbidity and mortality. Simple, inexpensive methods like adherence diaries to optimise effects of HAART would be useful.

**Methods:**

This quasi-experimental study was performed at a paediatric antiretroviral clinic in KwaZulu-Natal, South Africa. Children, from birth to 15 years, initiated on HAART from 01 August 2015 to 31 July 2016 were given illustrated medication diaries to be completed by caregivers. Viral load suppression and improvement in growth parameters and CD4+ percentage were determined at six months and one year. These outcomes were compared to those of a group of children who had been initiated on HAART from 01 August 2014 to 31 July 2015 and who had not received diaries.

**Results:**

Ninety-nine children were included in the historical control group and 35 children in the intervention group. Viral load suppression (HIV-1 RNA of < 400 copies/mL) was 72% in the control group and 71% in the diary group at 6 months (*p* = 0.6). At 12 months, 73% of children in the control group and 57% of the diary group had suppressed viral loads (*p* = 0.18). At 6 months, 63% of children in the control group and 57% of the diary group had improved weight for height *z*-scores (*p* = 0.09). At 12 months, when compared with baseline weight for height *z*-scores, there was improvement in 34% and 41% of the control and diary groups, respectively (*p* = 0.6). CD4+ percentages improved in 51% of the control group and 50% of the diary group at 6 months (*p* = 0.70); improvement was noted in 44% and 49%, respectively, at 12 months (*p* = 0.33).

**Conclusion:**

The addition of an illustrated medication diary to routine adherence counselling did not improve outcomes for children initiated on HAART.

## Introduction

Human immunodeficiency virus (HIV) and acquired immune deficiency syndrome (AIDS) are global issues, with substantial impact on the African continent. According to the Joint United Nations Programme on HIV and AIDS (UNAIDS) fact sheet on HIV and AIDS, in 2016, 36.7 million people across the world were HIV infected, with 2.1 million under the age of 15 years. The majority of HIV-infected individuals are found in middle- to low-income countries like South Africa. Fortunately, since 2000 there has been a steady increase in the number of people receiving antiretroviral therapy (ART). This number has increased from less than one million in 2000 to 18.2 million in 2016.^1^ However, despite increased awareness of the disease and available treatment, 40% of HIV-infected patients do not know their status.^2^ The consequences of HIV are far-reaching, affecting not only individual and public health but also economic growth and social stability. For example, it is estimated that there are 3.7 million AIDS orphans in South Africa.^3^

Highly active antiretroviral therapy (HAART) can help to prevent people living with HIV from developing AIDS and transmitting HIV.^4^ This is effective in suppressing HIV viral replication and improving immune system function in children.^5^ Early HIV diagnosis and early ART reduce early infant mortality by 76% and HIV progression by 75%.^6^ In 2013, the South African Department of Health released new antiretroviral guidelines that stated that all children under the age of five years should be initiated on HAART irrespective of their CD4+ count.^7^

The efficacy of HAART may be measured using HIV viral load and CD4+ counts.^8^ High levels of plasma HIV RNA are strongly predictive of clinical disease progression. Either a decrease in HIV RNA levels or an increase in CD4+ or both are associated with a delay in clinical disease.^9^ It has also been found that after 12 months of ART, 70% (95% confidence interval: 67–73) of patients have virologic suppression.^10^ ART also has a positive effect on height and weight of children with HIV infection.^11^

Good adherence to ART is associated with virologic response. Non-adherence to ART is a major public health concern as it leads to virologic, immunologic and clinical failure as well as increased transmission of drug-resistant virus.^12^ It is essential to achieve more than 95% adherence to ART regimens in order to suppress viral replication and to suppress emergence of resistance.^13^

Barriers to adherence to ART include the following:

1. **Patient-related:** fear of disclosure, depression or hopelessness, forgetfulness, reliance on caregiver, inconsistent caregiver and poor palatability of medication.
2. **Beliefs about medications:** side effects and unwanted changes in body image.
3. **Daily schedules:** complicated regimens, disruptions in routine and lack of convenience.

Facilitators of adherence include the following:

1. **Patient-related:** high self-worth, acceptance of HIV status, and supportive caregiver or family.
2. **Beliefs about treatment:** belief in efficacy and understanding the need for strict.
3. **Daily schedules:** compliance simple regimens and use of reminder tools.^14^

Medication diaries have been used to improve adherence to medication in a number of chronic disorders including rhinitis, migraines and haemophilia.^15,16,17^ Illustrated medication schedules, an easily understood tool to assist with medication management, have also been found to be useful.^18^ Persons of low literacy are more likely to miss treatment doses, suggesting that interventions are needed to help these persons adhere to antiretroviral therapies.^19^

Tuberculosis (TB) guidelines incorporate the use of a treatment card as part of directly observed treatment short-course (DOTS). The administration of therapy for *Mycobacterium tuberculosis* under direct observation leads to significant reductions in the frequency of acquired drug resistance and relapse.^20^ It has been found that guardians as well as healthcare workers are able to supervise TB treatment administration.^21^

A study performed in Kenya evaluated the use of medication diaries to improve outcomes with ART but did not find that adherence diaries were beneficial; however, it is of note that the study did not have adequate numbers to be conclusive.^22^ In another study conducted on a small group of non-responders, it was found that DOT had a positive impact, resulting in a significant drop in viral load.^23^

It is clear that despite improvements made in HIV prevention and care, HIV remains a major cause of morbidity and mortality. Antiretrovirals are effective in managing HIV and are now widely available, but adherence remains a problem. In this study, we tried to identify a simple, cheap and effective means to improve outcomes for children initiated on HAART by using an illustrated medication diary.

## Methods

### Study design

This is a quasi-experimental (before-after) study with illustrated medication diaries as the intervention.

### Setting

The study was conducted at Khanyisa Clinic, which is based at Edendale Hospital in Pietermaritzburg, KwaZulu-Natal. This paediatric antiretroviral clinic was established in 2007 and has a dedicated staff of nurses, counsellors and administration clerks. There are two permanent doctors in the clinic and a third doctor rotates through on a three-monthly basis.

### Participants

The study participants included all children who were initiated on first-line ART according to the South African National Department of Health antiretroviral guidelines from 01 August 2014 to 31 July 2016. Children who were initiated on HAART from 01 August 2014 to 31 July 2015 were included in the control group and those who were initiated on HAART from 01 August 2015 to 31 July 2016 were included in the diary group. Each group was followed up for one year after initiation.

Clinical protocols, laboratory monitoring, initiation of HAART, follow-up and adherence counselling were undertaken according to the existing practices at the clinic and followed the guidelines of the Department of Health. The exception to this was an additional CD4+ measurement taken at six months, which deviated from national guidelines.

### Diary use

Caregivers were given illustrated medication diaries that were to be completed each day. Verbal instructions were given to caregivers by the attending doctors working in the clinic. A copy of written instructions and information in both English and Zulu was provided to each caregiver at initiation. The diaries were in tabular form that required a tick to be made in the morning and evening when treatment was taken. Drugs and doses were listed on the diary as a reminder to the caregiver. On follow-up, doctors documented whether the diaries were complete or incomplete. If incomplete, the number of days missed was to be documented.

### Outcomes

Outcomes monitored included weight for height *z*-scores, CD4+ percentage categories, and HIV-1 RNA viral load at treatment initiation and after six months and 12 months of treatment. Weight for height *z*-scores was based on World Health Organization (WHO) *z*-score charts. CD4+ percentages were categorised into the following: ≤ 10%, 11% – 15%, 16% – 20% and > 20%. HIV-1 RNA viral load suppression was regarded as a viral load of < 400 copies/mL. Participants lost to follow-up were documented.

### Data analysis

Subgroup comparisons were made using Chi-square test or Fisher’s exact test for categorical variables. Numeric variables were compared using Wilcoxon rank sum test at each time point. Patients were also categorised into those who improved, those who declined and those who had no change in outcomes at six and 12 months when compared with baseline and compared between groups using Fisher’s exact test.

Data were analysed using STATA version 13 (*Stata Statistical Software: Release 13.* College Station, TX: StataCorp LP).

## Ethical consideration

Ethic approval was obtained from the University of KwaZulu-Natal Biomedical Research Ethics Committee (BREC). Site approval was obtained from the medical manager of Edendale Hospital. Department of Health approval was obtained from the provincial Department of Health. Consent was obtained from all caregivers participating in the study. Assent was obtained from all those children older than seven years who participated in the study. Names of the participants were not listed on the diary or during data collection

## Results

### Description of study participants

A total of 99 children were initiated on HAART from 01 August 2014 to 31 July 2015 and were included in the control group of the study. A total of 77 children were initiated on HAART from 01 August 2015 to 31 July 2016. Thirty-nine (51%) of these children received the diary. Four children were excluded from the study as they had previously defaulted treatment. A total of 35 participants were included in the diary group.

The median age of the participants was 28 months in the control group and 13 months in the diary group (*p* = 0.2). In the control group, 48% were females; in the diary group, 63% were females (*p* = 0.1).

At initiation, children in the diary group had non-statistically significant higher median CD4+ percentage (21.5% vs. 17.21% in the control group [*p* = 0.2]) and median HIV-1 RNA log viral load (12.6 vs. 11.8 in the control group [*p* = 0.6]). Distributions of HIV stage at initiation did not differ between the groups (*p* = 0.1).

At six and 12 months, 19 participants were lost to follow-up in the control group. At six months, 10 participants were lost to follow-up in the diary group, while at 12 months 11 participants were lost to follow-up. This resulted in groups of 80 and 24 children in the control and diary groups, respectively.

Baseline data for both groups are listed in Table 1.

**TABLE 1:** Baseline data for control and diary groups.

Patient characteristics	Baseline data											
	Control				Diary				Total			
	*n*	Median	%	IQR	*n*	Median	%	IQR	*n*	Median	IQR	*p*
Age	99	28	-	(7–84)	35	13	-	(5–60)	134	24	(6–72)	0.2
Weight	96	10.15	-	(5.7–18.3)	35	8.5	-	(4.6–14.5)	131	9,5	(5.6–17.5)	0.2
Height	89	82	-	(63–114)	35	68	-	(61–96)	124	79,6	(62–113)	0.1
CD4+%	94	17.21	-	(11.3–27.2)	33	21.5	-	(14.4–28.3)	127	18,4	(11.9–27.5)	0.2
Log viral load	92	11.8	-	(10.5–13.8)	30	12.6	-	(10.2–13.8)	122	12,2	(10.2–13.8)	0.6
**Sex**												
Male	51	-	51	-	13	-	37	-	-	-	-	0.1
Female	48	-	48	-	22	-	63	-	-	-	-	-
**HIV Stage**												
1	15	-	15	-	4	-	11	-	-	-	-	0.1
2	5	-	5	-	4	-	11	-	-	-	-	-
3	25	-	25	-	4	-	11	-	-	-	-	-
4	29	-	29	-	12	-	34	-	-	-	-	-
No stage	25	-	26	-	11	-	33	-	-	-	-	-
**Weight for height**												
−3 *z*-score	17	-	17	-	9	-	26	-	-	-	-	-
−2 *z*-score	12	-	12	-	4	-	11	-	-	-	-	-
−1 *z*-score	19	-	19	-	4	-	11	-	-	-	-	-
0 *z*-score	18	-	18	-	5	-	14	-	-	-	-	-
+1 *z*-score	18	-	18	-	6	-	17	-	-	-	-	-
+2 *z*-score	3	-	3	-	3	-	9	-	-	-	-	-
+3 *z*-score	2	-	2	-	4	-	11	-	-	-	-	-
No *z*-score	10	-	10	-	0	-	0	-	-	-	-	-
**CD4+ category**												
< 10%	19	-	19	-	5	-	14	-	-	-	-	-
10–15	22	-	22	-	5	-	14	-	-	-	-	-
16–20	15	-	15	-	5	-	14	-	-	-	-	-
> 20	38	-	38	-	18	-	51	-	-	-	-	-
No CD4+%	5	-	5	-	2	-	6	-	-	-	-	-

IQR, interquartile range.

### Virologic response

Human immunodeficiency virus-1 RNA viral loads were available for 72 children (73%) in the control group and 21 children (60%) in the diary group at six months. At 12 months, viral loads were available for 71 children (72%) in the control group and 21 children (60%) in the diary group.

**FIGURE 1:**
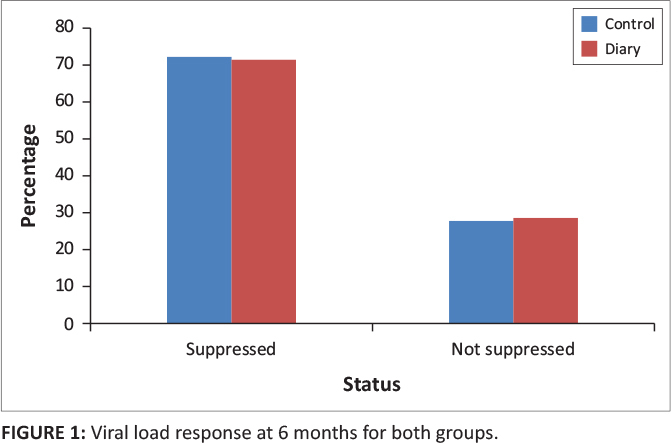
Viral load response at 6 months for both groups.

At six months, there were 52 children (72%) in the control group with suppressed viral load and 15 children (71%) in the diary group with suppressed viral load (*p* = 0.6) (Table 2 and Figure 1).

**TABLE 2:** Viral load response at 6 and 12 months for both groups.

Status	Viral load (log)				*p*
	Control		Diary		
	*n*	%	*n*	%
**6 Months**					
Suppressed	52	72	15	71	0.6
Not suppressed	20	28	6	29	
**12 Months**					
Suppressed	52	73	12	57	0.18
Not suppressed	19	27	9	43	

**FIGURE 2:**
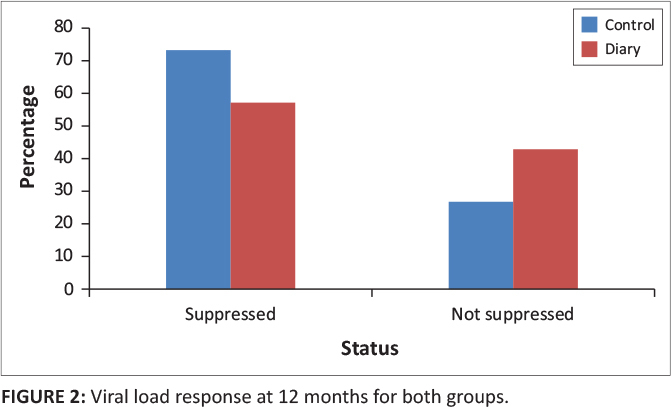
Viral load response at 12 months for both groups.

At 12 months, there were 52 children (73%) in the control group and 12 children (57%) in the diary group who had suppressed HIV viral load (*p* = 0.18) (Table 2 and Figure 2).

### Immunologic response

CD4+ percentages were available for 61 children (76%) in the control group and 20 children (80%) in the diary group at six months and for 61 children (76%) in the control group and 16 children (67%) in the diary group at 12 months.

**FIGURE 3:**
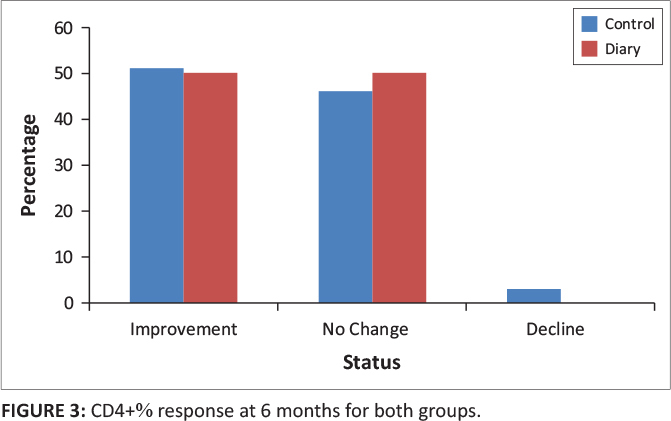
CD4+% response at 6 months for both groups.

At six months, in the control group, 31 participants (51%) had an improvement in CD4+ percentage category, 28 (46%) had no change in percentage category and two (3%) had a decline in CD4+ percentage category. In the diary group, 10 participants (50%) had an improvement in CD4+ percentage category, 10 (50%) had no change in percentage category and none of the participants had a decline in CD4+ percentage category (*p* = 0.70) (Table 3 and Figure 3).

**TABLE 3:** CD4+% response at 6 and 12 months for both groups.

Status	CD4+%				*p*
	Control		Diary		
	*n*	%	*n*	%
**6 months**					
Improvement	31	51	10	50	0.7
No change	28	46	10	50	
Decline	2	3	0	0	
**12 months**					
Improvement	31	51	6	38	0.4
No change	28	46	8	50	
Decline	2	3	2	13	

**FIGURE 4:**
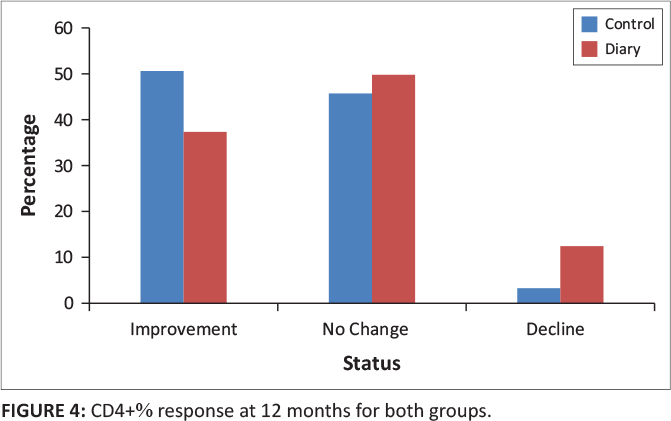
CD4+% response at 12 months for both groups.

At 12 months, in the control group, 31 participants (51%) had an improvement in CD4+ percentage category, 28 (46%) had no change in percentage category and two (3%) had a decline in CD4+ percentage category. In the diary group, six participants (38%) had an improvement in CD4+ percentage category, eight (50%) had no change in percentage category and two (13%) participants had a decline in CD4+ percentage category (*p* = 0.33) (Table 3 and Figure 4).

### Growth parameters

Weight for height *z*-scores was collected at initiation, six months and 12 months. Baseline weight for height *z*-scores was available for 89 children (90%) in the control group and 34 children (97%) in the diary group. The *z*-scores ranged from -3 to +3. An improvement in *z*-score was defined as a positive change along this range. A change from 1 *z*-score to a higher *z*-score (e.g. from +1 to +2) was regarded as a relevant positive change.

**FIGURE 5:**
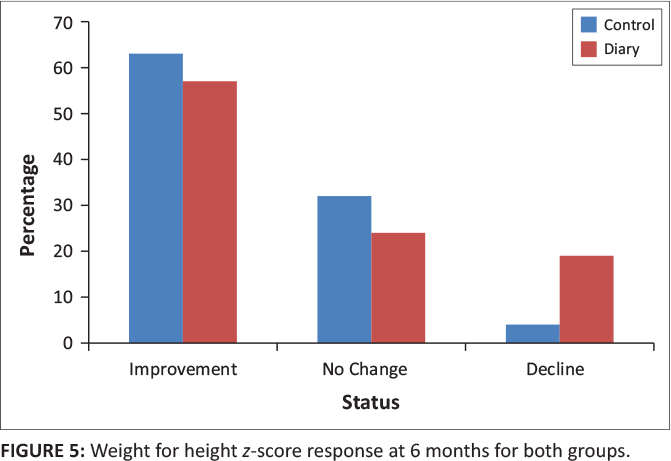
Weight for height *z*-score response at 6 months for both groups.

At six months, weight for height *z*-scores were available for 68 (85%) out of 80 children in the control group and 21 (84%) out of 25 children in the diary group. Forty-three (63%) children in the control group had improved weight for height *z*-scores, whereas 12 (57%) children in the diary group had improved weight for height *z*-scores. Twenty-two (32%) children in the control group had no change in weight for height *z*-scores and three (4%) had worsening *z*-scores. Five (24%) children in the control group had no change in weight for height *z*-scores and four (19%) had worsening *z*-scores (*p* = 0.09) (Table 4 and Figure 5).

**TABLE 4:** Weight for height *z*-score response at 6 and 12 months for both groups.

Status	Weight for height *z*-scores				*p*
	Control		Diary		
	*n*	%	*n*	%
**6 months**					
Improvement	43	63	12	57	0.09
No change	22	32	5	24	
Decline	3	4	4	19	
**12 months**					
Improvement	42	66	8	44	0.17
No change	17	27	5	28	
Decline	5	8	5	28	

**FIGURE 6:**
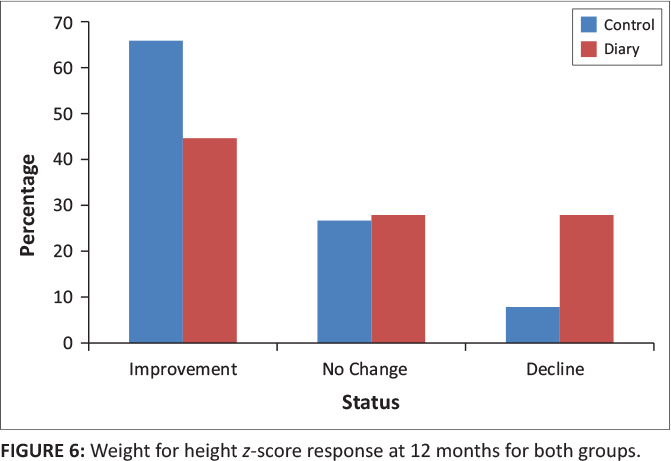
Weight for height *z*-score response at 12 months for both groups.

At 12 months, weight for height *z*-scores was available for 64 (80%) out of 80 children in the control group and 18 (75%) out of 24 children in the diary group. Forty-two (66%) children in the control group had improved weight for height *z*-scores, whereas eight (44%) children in the diary group had improved weight for height *z*-scores. Seventeen (26%) children in the control group had no change in weight for height *z*-scores. Five (8%) children had worsening *z*-scores. Five (28%) children in the control group had no change in weight for height *z*-scores and five (28%) had worsening *z*-scores (*p* = 0.17) (Table 4 and Figure 6).

### Lost to follow-up

At six months, 19 (19%) participants in the control group and 10 (28%) in the diary group were lost to follow-up. At 12 months, a further one participant was lost to follow-up from the diary group. The national laboratory data base was searched using the names and dates of birth of these children, and no record was found after their initiation or last follow-up at the clinic. One patient in the intervention group defaulted treatment at six months and was reinitiated on HAART after two months. One patient in the control group was documented as demised.

## Discussion

In this quasi-experimental study, the use of illustrated medication diaries did not improve virologic, immunological or clinical outcomes further than existing practices for children initiated on HAART.

The results of our study are in agreement with a similar study conducted in Kenya in 2011. This randomised control trial compared outcomes in children receiving HAART who had received conventional counselling with those who received a medication diary. The investigators concluded that medication diaries were not beneficial. The study did not have adequate numbers to be conclusive, and there were a large number of participants who were lost to follow-up.^22^ In contrast, another study that was conducted on a small group of children who had a poor response to therapy found that DOT leads to a decrease in viral load. Children who had poor responses to ART with high viral loads despite repeated caretaker reports of good adherence to treatment were included in the study. The participants were admitted to the hospital and had a period of 4–8 days of documented medication administration. Nursing staff observed patients while medication was ingested. A diary was not included as part of the DOT. Plasma viral loads at the times of admission and discharge were measured to determine whether the short period of DOTS had an effect on viral load.^23^

We have identified potential reasons for the negative findings of our study. A few differences in baseline characteristics were noted between the control and diary groups, although these were not found to be statistically significant. The median viral load of children in the diary group was higher than that of the control group. A greater proportion of participants in the diary group were categorised as stage 4 HIV and were severely malnourished with weight for height *z*-scores less than -3. This suggests that participants in the diary group were possibly more ill than those in the control group, although the number of participants did not provide sufficient power to demonstrate this statistically.

Another notable difference in both groups was the median age. Participants in the diary group were younger than those in the intervention group. According to national guidelines, patients under the age of three years and under 10 kg receive lopinavir/ritonavir as part of the first-line regimen.^7^ A known characteristic of protease inhibitors such as lopinavir and ritonavir is the bitter taste.^24^ Poor palatability has been found to adversely affect adherence to ART.^25^ Adherence to syrup formulations, which are more commonly used in younger children, has also been found to be lower than for tablets. This was the result found in a study in Uganda – median adherence to syrup formulations (97%, IQR [interquartile range] 93–98) was significantly lower than for tablets (100%, IQR 97–100, *p* = 0.012, *n* = 28) using pharmacy refill data.^26^ In our study, the diary group had a younger median age which could have adversely affected outcomes for these reasons.

We have identified a few limitations of this study that may explain the lack of efficacy of the diaries in improving outcomes. These include the following:

1. **Inadequate numbers in the diary group:** A total of 77 children were initiated on HAART from 01 August 2015 to 31 July 2016. Only 39 of these patients received the diaries. There were multiple staff-related reasons for this, including lack of cooperation from some staff members in the clinic; staff shortages and related time constraints that precluded extra time being taken to explain the study and obtain consent or assent; and lack of awareness of the study because of rotation of doctors. There was only one documented case of a parent refusing to participate in the study. Attempts to overcome the above included in-service training of permanent staff in the clinic, posters in the consultation rooms to remind staff about the diaries, packs that contained all necessary paperwork for the study (the diaries, consent and assent forms and information handouts) to make it quicker and easier to enrol patients, and involvement of the counsellors to introduce the concept of the diaries during the compulsory adherence classes prior to starting antiretrovirals.
2. **Poor documentation of the use of the diary:** This again was a staff-related factor and the reasons included lack of cooperation, lack of staff numbers and lack of awareness of the diaries. Of the 35 children who were included in the diary group, only four had documentation of diary use. Two children had documentation of the diary being complete and two children had lost the card and were given replacement cards.
3. **Pill counts were not performed:** Pill counts were not routinely performed at the clinic. Pill counts could have been used to correlate the use of the diary with level of adherence to treatment and treatment outcomes. There was no way of knowing whether the caregivers completed the diaries just before the appointment date or completed the diary on a daily basis as they were instructed.
4. **Medication shortages and changes:** During the diary intervention period, there was a country-wide shortage of abacavir. This meant that the medication regimens of all participants had to be changed to one that did not include abacavir according to the guidelines released by the Department of Health. When abacavir became available, regimens were changed back to the original regimen. This may have caused some confusion to caregivers or patients and affected outcomes.
5. **Fear of disclosure:** Names were not listed on the diaries in order to avoid unplanned disclosure. However, the diaries listed the treatment on the back cover, which may have led the caregivers to fear of accidental disclosure if diaries were found in their possession by others.
6. **Lack of blood results:** A number of patients did not have results for CD4+ and viral loads in both the diary and intervention groups. It was a common practice for caregivers to collect medication for their children each month without the children being present. Laboratory specimens were sometimes rejected because of insufficient samples or incorrect tubes being used – the practice of caregivers collecting medication for children made it difficult to correct such mistakes until months later. Staff-related issues such as rotating staff, which included more junior staff who did not have sufficient knowledge of laboratory monitoring in these patients, as well as skills to collect adequate blood specimens contributed to this. Additionally, feedback to patients and caregivers is based on clinical and laboratory findings, and if blood results are unavailable, this could compromise patient and caregiver education and counselling.

During our study, we observed that the majority of patients had improved growth parameters, CD4+ percentages and HIV viral loads, regardless of having received the diary or not. This suggests that the existing measures, like adherence counselling to improve outcomes of children receiving HAART, are generally effective. However, there were participants in both groups who did not have favourable outcomes, including loss to follow-up, suggesting that methods to improve adherence to HAART and to maximise the effect of HAART continue to be warranted. Further studies are required to identify ways to do so.
